# Knowledge, Attitude and Practices Towards Dengue Fever Among Slum Dwellers: A Case Study in Dhaka City, Bangladesh

**DOI:** 10.3389/ijph.2023.1605364

**Published:** 2023-05-22

**Authors:** Md. Mostafizur Rahman, Kamrun Nahar Tanni, Tuly Roy, Md. Rakibul Islam, Md. Alim Al Raji Rumi, Mohammed Sadman Sakib, Masrur Abdul Quader, Nafee-Ul-Islam Bhuiyan, Ifta Alam Shobuj, Afra Sayara Rahman, Md. Iftekharul Haque, Fariha Faruk, Fahim Tahsan, Farzana Rahman, Edris Alam, Abu Reza Md. Towfiqul Islam

**Affiliations:** ^1^ Department of Disaster Management and Resilience, Faculty of Arts and Social Sciences, Bangladesh University of Professionals, Dhaka, Bangladesh; ^2^ Department of Disaster and Human Security Management, Faculty of Arts and Social Sciences, Bangladesh University of Professionals, Dhaka, Bangladesh; ^3^ Department of Computer Science and Engineering, Independent University, Bangladesh, Dhaka, Bangladesh; ^4^ Faculty of Resilience, Rabdan Academy, Abu Dhabi, United Arab Emirates; ^5^ Department of Geography and Environmental Studies, University of Chittagong, Chittagong, Bangladesh; ^6^ Department of Disaster Management, Begum Rokeya University, Rangpur, Bangladesh

**Keywords:** COVID-19, Bangladesh, slums, dengue, Dhaka city

## Abstract

**Objectives:** This study intends to evaluate Dhaka city slum dwellers’ responses to Dengue fever (DF).

**Methods:** 745 individuals participated in a KAP survey that was pre-tested. Face-to-face interviews were performed to obtain data. Python with RStudio was used for data management and analysis. The multiple regression models were applied when applicable.

**Results:** 50% of respondents were aware of the deadly effects of DF, its common symptoms, and its infectious nature. However, many were unaware that DF could be asymptomatic, a previously infected person could have DF again, and the virus could be passed to a fetus. Individuals agreed that their families, communities, and authorities should monitor and maintain their environment to prevent *Aedes* mosquito breeding. However, overall 60% of the study group had inadequate preventative measures. Many participants lacked necessary practices such as taking additional measures (cleaning and covering the water storage) and monitoring potential breeding places. Education and types of media for DF information were shown to promote DF prevention practices.

**Conclusion:** Slum dwellers lack awareness and preventative activities that put them at risk for DF. Authorities must improve dengue surveillance. The findings suggest efficient knowledge distribution, community stimulation, and ongoing monitoring of preventative efforts to reduce DF. A multidisciplinary approach is needed to alter dwellers’ behavior since DF control can be done by raising the population’s level of life. People and communities must perform competently to eliminate vector breeding sites.

## Introduction

Dhaka, the capital city of Bangladesh, has had a rapid population increase and density [1]. Migration caused by climate change has exacerbated these concerns [2]. Dengue fever (DF) and the COVID-19 pandemic have reached alarming levels in the city [3–9]. DF, a neglected tropical disease, wreaks havoc on developing countries, particularly in Asia [10, 11]. It was initially identified in 1780, but the first occurrence of dengue in Bangladesh was reported in 1964 under the name “Dhaka/Dacca fever” [12]. Dengue cases were then intermittently recorded in Bangladesh, primarily in Dhaka [12]. Additionally, a rapid spike in dengue cases was seen after the year 2000, with 2019s large number of infections resulting in several fatalities [13]. It has been a burden on the country, particularly Dhaka, which was severely affected by a rapid dengue outbreak [3]. This densely populated metropolis accounted for over half of the country’s dengue cases in 2019 [13]. Studies indicate that a high population density, increasing unplanned development, limited dengue surveillance operations, contempt for dengue protective behavior, and climate change might all lead to a significant dengue outbreak in the city [14, 15]. In addition, research predicted that this city would be the core of the country’s significant dengue outbreaks [16]. Moreover, according to research, the rapid development of the dengue outbreak across the country in 2019 was driven by the huge migration of dengue-infected individuals (many of whom were anticipated to be asymptomatic) from Dhaka to other cities in the country [3]. It is considered that Bangladesh’s ailing healthcare system has led to a considerable underreporting of Dengue cases [17]. Individuals are concerned about the impact of the COVID-19 pandemic because the city has already been severely affected. Even though dengue and COVID-19 include fever symptoms and asymptomatic cases, the number of reported dengue cases during the pandemic cannot be ignored [18]. Consequently, local inhabitants have battled to limit the disease’s catastrophic effects.

While there is no effective vaccination for DF, health habits such as adherence to accurate knowledge, having a good attitude, and sticking to safe practices may pave the road for DF eradication [19]. Changes in individual and community behavior, as well as appropriate government support for DF can help reduce the growing prevalence of dengue in Dhaka and, by extension, throughout the country. DF has already been the subject of several studies in Bangladesh [3, 6, 15, 20]. However, knowing the resident’s knowledge, attitude, and practice (KAP) of DF is critical for efficient vector-borne disease control [21]. The KAP model, developed in the 1950s, has been widely used to assess survey respondents’ understanding of a subject [8, 9, 22, 23]. The simple design, normative data, and precise report make this survey approach convenient [24]. Additionally, it is assumed to demonstrate the interaction between respondents’ KAP domains [25]. Communities may be better able to prepare for and respond to public health emergencies if the KAP level is known. In addition, such research may help to create interventions that encourage desired behavioral changes [26]. Several web-based research of KAP among Dhaka’s general population and university students showed a statistically significant correlation between socio-demographic data and KAP about DF [6, 20]. However, despite the increased risk of DF among slum dwellers [27–29], there is a lack of research on KAP concerning DF in Dhaka’s slums. According to the KAP survey, we can assess the level of DF preparedness for this vulnerable group. The findings can assist the policies and strategies that reduce the risk of DF for these vulnerable people.

Today’s megacities are extremely diverse, with substantial slum populations posing issues for community health and healthcare [30]. Depending on where you reside in a major urban area, there might be vast variances in health conditions. In general, urban health is better than rural health, but in particular areas, urban health might be poorer [31]. Certain infectious diseases have emerged and re-emerged due to the urban environment’s constant change. Pathogens adapted to urban conditions from rural environments can spread rapidly and place a higher load on healthcare systems [32]. Bangladesh’s slums are densely populated urban areas with substandard housing and squalor. According to the findings of a study, slum areas are afflicted with DF [27]. The density of *Aedes aegypti* mosquito larvae has been found to be four times higher than the normal level in the slums of the city [28, 29]. Moreover, given the recent increase in dengue mortality inside Dhaka, it is essential to evaluate the dengue-associated KAP of city slum dwellers to establish efficient vector control and monitoring techniques. This study aimed to assess the KAP towards DF among Dhaka’s slum dwellers. This study may provide international, national, and municipal authorities, as well as non-governmental and social groups regulating the DF with crucial baseline data.

## Methods

### Study Design and Population

During the COVID-19 pandemic, a cross-sectional study was performed. The study is delimited to Dhaka, the city with the most slum residents in Bangladesh [33]. This study was conducted as part of a research project approved by the Research Ethics Committee of Bangladesh University of Professionals, Dhaka, Bangladesh (BUP REC0907/2019). It evaluated all relevant ethical concerns. The aims and ethical problems of the survey were articulated on the questionnaire’s cover page. Consent was obtained from respondents, who remained anonymous.

**FIGURE 1:**
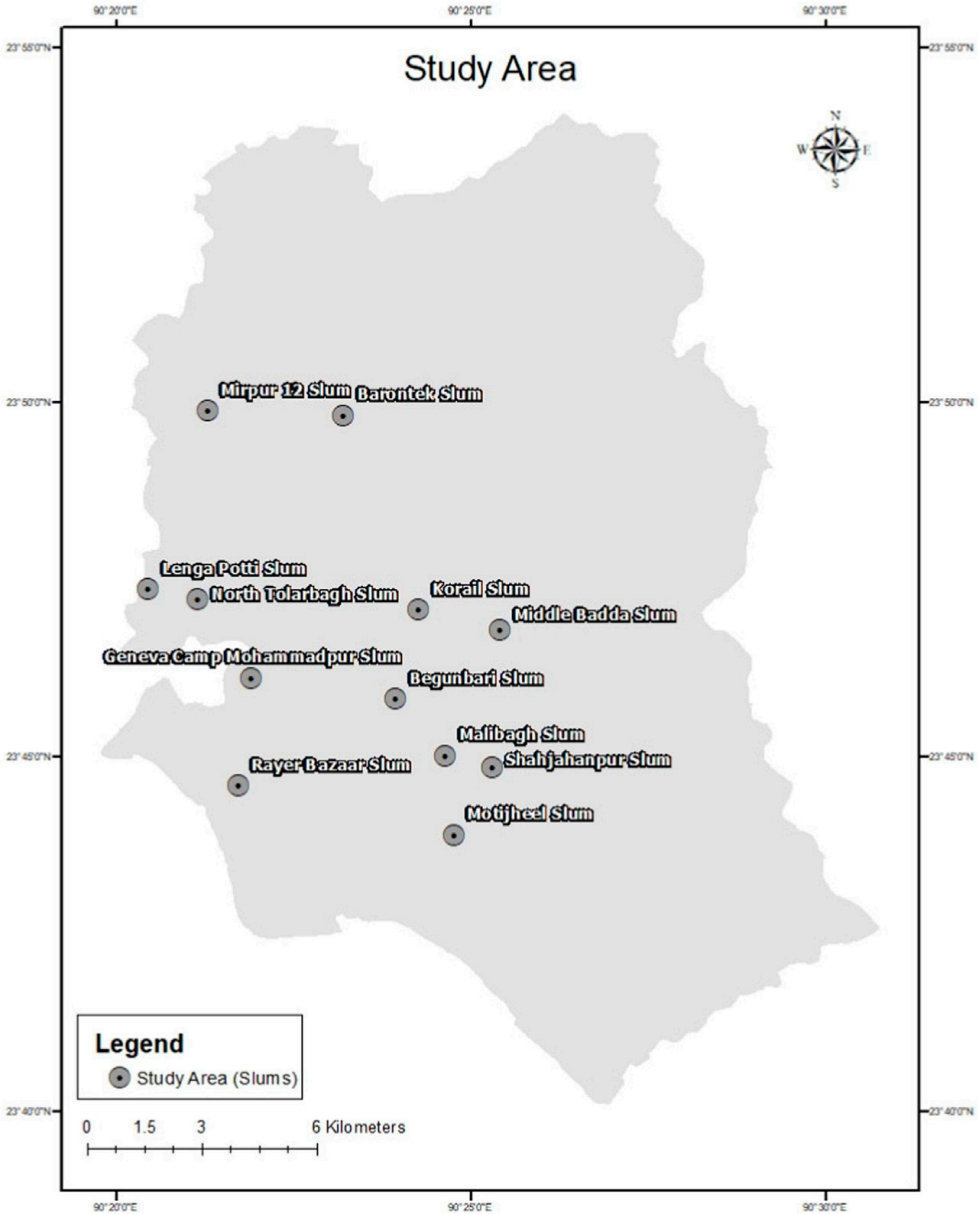
The study area (Dhaka, Bangladesh. 2021).

Over one-third of Dhaka’s population lives in one of the city’s 5,000 slums or informal settlements, and slum populations are rising at more than double the rate of urban areas overall [33]. One study indicated that more than 37.0% of the population of this city lives in slums [34]. Slums usually have no access to piped water and temporary containers like drums and earthen jars commonly store water where *Aedes* mosquito lays eggs [35]. Inadequate supplies of piped water and an absence of proper waste management in most locations of Dhaka result in abundant indoor and outdoor mosquito breeding sites [36]. Most slum dwellers are immigrants living in economically depressed conditions [37]. In addition to lacking permanent work, their health and physical conditions are substandard. We have surveyed 12 slums in Dhaka city (Figure 1).

### Questionnaire

The Bangladeshi viewpoint was considered when adapting and developing the draft questionnaire based on a thorough review of prior research [15, 21, 38–41]. In order to pre-test the questionnaire, experts’ comments and a pilot survey were conducted after that. A final questionnaire was prepared with slum dwellers in consideration. The survey was administered in Bengali, the local language. It consisted of two primary elements: respondent information and KAP sections. There were 32 items in the KAP section. There have been 13 closed-ended questions in the knowledge section (DF is an infectious disease, it can cause death, common symptoms of the disease, vector type, phenotype of the mosquito, breeding sites of the mosquito, biting time, DF risk during pregnancy, and DF infection characteristics). The scoring range was from 0 to 1, with the “Maybe” option (if applicable) receiving a score of 0.50. The attitude section contained 9 closed-ended items with a 5-point Likert scale (Strongly agree = 5, Agree = 4, Neutral = 3, Disagree = 2, and Strongly disagree = 1). It had items such as the responsibility to ensure the absence of vector mosquitoes and breeding sites; Regular DF surveillance activities; Community commitment to control the outbreak; Participation in DF control public activity; Taking immediate treatment; and Concerning DF. The final practice section contained 10 items with Yes/No answers and a 0–1 score range (communicating the authority for fogging, using mosquito repellent and coil or mosquito-killing tools, checking the mosquito breeding sites, covering and cleaning the water containers, cleaning the plant pots, visiting a hospital for treatment, and following good practices during a pandemic). We calculated Cronbach’s alpha for internal consistency in the KAP domain, where knowledge = 0.84, attitude = 0.88, practice = 0.67, and overall KAP = 0.87. Cronbach’s alpha >0.60 suggests an adequate level, whereas >0.80 implies an outstanding one [42].

### Sampling Design

We have followed the non-probability, purposive, and convenience sampling techniques. Convenience sampling is a non-probability sampling technique in which units are included in the sample because they are the most accessible to the researcher [43]. It may be due to geographical accessibility, availability at a specific moment, or a willingness to engage in the study. First, we chose a slum based on its accessibility (to people and known persons who live in slums) and DF risk (based on previous reports). For instance, the disease control section of the Directorate General of Health Services of Bangladesh performed a survey from the end of July to the beginning of August 2019, and they found that the number of *Aedes* mosquito larvae was four times higher than they had anticipated at the Shajahanpur slum [28]. In our prior study on fire preparedness in Dhaka’s slums, we employed a similar sampling technique [44]. We only selected individuals over 18 years who resided in a particular slum and were accessible. Finally, we maintained a minimum number of participants (around 50) from each of our selected slums. The sample size was calculated using Yamane’s formula [45]:

$$n=\frac{N}{1+N\left(e^{2}\right)}$$

where n = sample size, N = population, e = error tolerance.

Approximately 4 million slum dwellers were found in Dhaka city [33]. Therefore, following this population and 0.05 error tolerance, the required sample size was around 400.

### Data Collection

The survey was conducted from January to March of 2021. There was a face-to-face interview. Consent was taken. The data was double-checked for anomalies.

### Data Management and Analysis

Python (version 2.7; Beaverton, OR 97008, United States) and RStudio (version 1.2.5042; Boston, MA, United States) [46, 47] were used for data analysis and management. All statistical analysis was performed with 95% CI. When applicable, descriptive analyses (frequency, percentage, mean, and standard deviation) were calculated. First, the average score was calculated to ensure that the same scale was utilized throughout the study. It was accomplished by first computing the section’s overall score by adding the component scores. The section’s mean score was then calculated by dividing the total score by the number of items in the section (Eq. 1). In the case of attitude, this number is again divided by 5 (this section had 5 scales). Consequently, all three portions were scaled the same (0–1 score range).

$$average\;score\;of\;dimension=\frac{\sum score\;in\;items}{\sum item\;number}$$ (1)

$$Knowledge=\sum_{1}^{13}\frac{S_{i}}{n}$$

$$Attitude=\sum_{1}^{09}\frac{S_{i}}{n\times 5}$$

$$Practice=\sum_{1}^{10}\frac{S_{i}}{n}$$

Where *i* is for *ith* item’s score and *n* is the total number of items.

Spearman’s rank correlation was applied to assess the correlation in the KAP domain. In multiple linear regression analyses, predictors (*p* < 0.05) from univariate linear regression analyses were used after screening.

## Results

### Respondent’s Characteristics

We examined 745 responses in the final analysis. Table 1 reveals that most respondents were around 18–35 years (around 70%). The male respondents (54%) were more than the female respondents (46%). Most respondents were married (71%) and living with their families (84%). More than 50% did not have any education. Many indicated they have limited income sources, such as day workers, rickshaw pullers, vehicle drivers, home maids, etc. Therefore, we considered them workers with limited income compared to others without any earning source. Regarding preferred media for DF information, they primarily used electronic media such as Television, Radio (48%), and their family members or community (36%).

**TABLE 1:** Characteristics of the respondents (*n* = 745) (Dhaka, Bangladesh. 2021).

Characteristics	n (%)
1. Age (year)	
18–25	265 (35.57)
26–35	276 (37.05)
36–45	131 (17.58)
More than 45	73 (9.80)
2. Gender	
Male	402 (53.96)
Female	343 (46.04)
3. Marital status	
Married	527 (70.74)
Unmarried	132 (17.72)
Other (Separated, Divorced)	86 (11.54)
4. Living with family	
Yes	627 (84.16)
No	118 (15.84)
5. Education	
≥ Primary level	363 (48.72)
No education	382 (51.28)
6. Occupation	
Worker	533 (71.54)
No work	212 (28.46)
7. COVID-19 has negative impact on dengue fever surveillance activities	
Yes	237 (31.81)
Maybe	356 (47.79)
No	152 (20.40)
8. Media used for dengue fever information	
Electronic media (Television, Radio)	361 (48.46)
Social media	94 (12.62)
People (community, family members)	269 (36.11)
Others	21 (2.82)

### Knowledge Regarding Dengue Fever

The knowledge, attitudes, and practices about DF are displayed in Table 2. Over half of the slum dwellers (57%) knew that dengue is an infectious disease. In comparison, around 54% were aware of the common symptoms of dengue fever, including rash, headache, high fever, joint pain, muscle pain, nausea, etc. Only 26% knew of the asymptomatic or mildly symptomatic DF cases. Additionally, almost 60% of them were aware of deaths caused by the DF. However, few (29%) indicated familiarity with the DF situation for multiple occurrences in the same individual, and fewer (23%) were aware that DF could be severe the second time. Additionally, roughly 19% knew the dengue virus could be transmitted from an infected pregnant woman to the fetus. Approximately 40% of the participants knew the likely breeding location and frequency of insect bites. However, only 11% knew the number for the health call center.

**TABLE 2:** Knowledge, attitude, and practices towards dengue fever (Dhaka, Bangladesh. 2021).

Knowledge								
No	Items			Correct response [*n* (%)]			
K1	Dengue is an infectious disease			422 (56.64)				
K2	Dengue fever can cause death			445 (59.73)				
K3	Common symptoms of dengue infection are rash, headache, high fever, joint pain, muscle pain, nausea			400 (53.69)				
K4	People can have dengue virus even without any (or mild symptoms) symptoms			197 (26.44)				
K5	One person can be infected with dengue virus more than once			214 (28.72)				
K6	Second time dengue infection can be severe			168 (22.55)				
K7	Dengue virus can be transmitted from infected pregnant mother to fetus			143 (19.19)				
K8	Aedes mosquito type by which dengue virus is transmitted			377 (50.60)				
K9	Aedes mosquito has stripes on the body			379 (50.87)				
K10	I know the breeding place of Aedes mosquitoes			312 (41.88)				
K11	I know that Aedes mosquito breed both indoor and outdoor			317 (42.55)				
K12	I know that Aedes mosquito usually bites early in the morning and late evening			289 (38.79)				
K13	I know health call center number of the authority			79 (10.60)				
**Attitude**								
**No**	**Items**	**^a^SA [n (%)]**	**^a^A [*n* (%)]**		**^a^N [*n* (%)]**	**^a^DA [*n* (%)]**		**^a^SDA [*n* (%)]**
A1	It is my obligation to ensure that there are no *Aedes* eggs or larvae in the vicinity of my home	95 (12.75)	238 (31.95)		224 (30.07)	180 (24.16)		08 (1.07)
A2	My relatives and neighbors should clean *Aedes* mosquito breeding sites weekly, such as water containers, storage tanks, and plant pots	73 (9.80)	277 (37.18)		230 (30.87)	159 (21.34)		06 (0.81)
A3	Only chemical fogging by the authority is insufficient to prevent dengue infection; the authority must also destroy possible breeding grounds	94 (12.62)	292 (39.19)		225 (30.20)	124 (16.64)		10 (1.34)
A4	We should routinely examine the dengue situation or hotspots in our neighborhood	80 (10.74)	268 (35.97)		212 (28.46)	175 (23.49)		10 (1.34)
A5	Even when an outbreak is not occurring, it is vital to maintain eliminating mosquito breeding grounds	75 (10.07)	294 (39.46)		217 (29.13)	148 (19.87)		11 (1.48)
A6	Dengue outbreak in my area may be contained if every family removes mosquito breeding grounds	72 (9.66)	292 (39.19)		210 (28.19)	162 (21.74)		09 (1.21)
A7	I will participate in a public action for dengue control or mosquito breeding place elimination	72 (9.66)	215 (28.86)		234 (31.41)	204 (27.38)		20 (2.68)
A8	If a member of my family exhibits symptoms of dengue fever, I will bring him or her to a doctor immediately for treatment	76 (10.20)	323 (43.36)		209 (28.05)	128 (17.18)		09 (1.21)
A9	I am concerned about dengue even in the Coronavirus pandemic time	77 (10.34)	275 (36.91)		244 (32.75)	138 (18.52)		11 (1.48)
**Practices**								
**Items**				**Yes [*n* (%)]**			**No [*n* (%)]**	
P1	I call Municipality authority for fogging			29 (3.89)			716 (96.11)	
P2	I follow different ways (aerosol and/or liquid mosquito repellent and/or mosquito coil and/or electrical mosquito mat and/or mosquito bed net) to reduce mosquito			613 (82.28)			132 (17.72)	
P3	I wear proper cloth to protect from mosquito biting			149 (20.00)			596 (80.00)	
P4	I take extra precautions when I travel, to protect from mosquito biting			94 (12.62)			651 (87.38)	
P5	I properly cover water containers used for water storage			399 (53.56)			346 (46.44)	
P6	I keep the water-holding containers (tires, plastic bottles, parts of automobiles, cracked pots, plant pots) clear and drain the extra water			301 (40.40)			444 (59.60)	
P7	I scrub and clean the inner sides of the containers			273 (36.64)			472 (63.36)	
P8	I check for the presence of Aedes eggs and/or larvae inside or outside the house			173 (23.22)			572 (76.78)	
P9	I visit hospital for test and treatment when I see symptoms of Dengue			424 (56.91)			321 (43.09)	
P10	I follow this disease reduction practices even in the Coronavirus pandemic			499 (66.98)			246 (33.02)	

^a^ SA, strongly agree; A, Agree; N, Neutral; DA, Disagree and SDA, strongly disagree.

Overall, 50% of respondents replied to appropriate answers to questions regarding the deadly effects of DF, common symptoms of DF, and the infectious nature of DF. In addition, around half of them recognized that the female *Aedes* mosquito transmits the dengue virus and has a striped body. However, many respondents were unaware that this disease might be asymptomatic (a person could have it without or with mild symptoms), that a previously infected person could have DF again, which could be more severe, and that the virus could be passed to a fetus.

### Attitude Regarding Dengue Fever

Table 2 reveals that around 55% of respondents did not agree or were neutral on whether or not they were obligated to guarantee that there are no *Aedes* eggs or larvae near their dwellings. In addition, many respondents (52%) disagreed or were neutral about the weekly elimination of *Aedes* mosquito breeding areas. However, almost 50% of respondents agreed they should continue eliminating mosquito breeding areas even during the off-season. Furthermore, 51% stated that chemical fogging alone is insufficient to prevent dengue transmission; authorities must also eliminate potential breeding sites. Less than half (38%) agreed to participate in public action for dengue control or eradication of mosquito breeding places.

Overall, individuals agreed that their families, adjacent communities, and the appropriate authorities are responsible for eliminating potential *Aedes* mosquito breeding places and guaranteeing the absence of *Aedes* mosquito eggs and larvae in their immediate environment. They agreed that even if the outbreak is not evident, these activities should be regularly monitored and maintained. Some people thought they were obligated to participate in public DF control programs. Even throughout the COVID-19 outbreak, they were concerned for DF.

### Practices Regarding Dengue Fever

Table 2 demonstrates that only 4% of participants contact the municipality for fogging. Approximately 82% of respondents reported utilizing mosquito control or removal techniques. We have also asked about the tools used to protect from mosquito bites for further investigation. Many of them used bed nets (55%) and mosquito coils (44%). They did not, however, cover the water storage containers (46%) or clean them (60%). They go to a hospital for DF testing and treatment (57%). Few of them (23%) monitor the presence of *Aedes* eggs and/or larvae within and around their dwellings. Even during the pandemic, around 67% adhered to DF precautions.

Overall, the vast majority of participants (>70%) lacked necessary practices, such as engaging with local authorities for fogging, taking additional measures (wearing appropriate clothes, etc.) for DF, and monitoring potential *Aedes* mosquito breeding places.

### Correlation in KAP Domain

**FIGURE 2:**
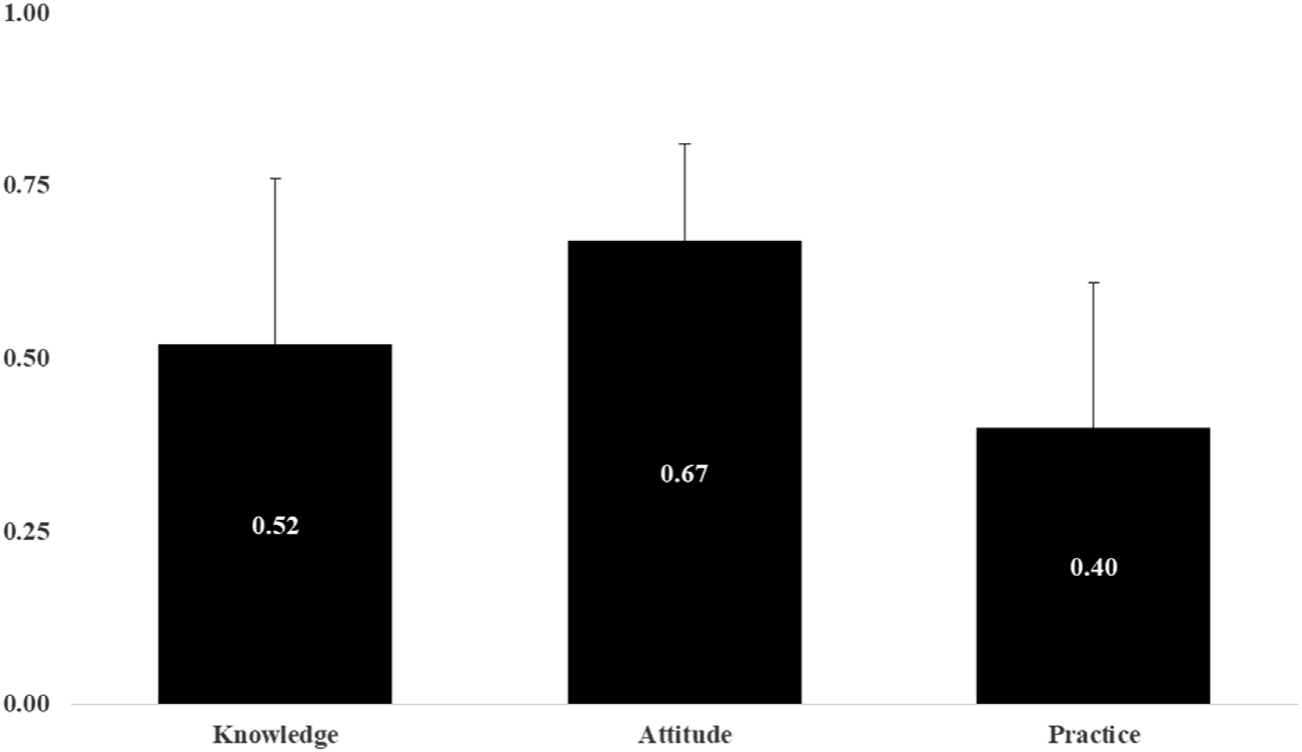
Mean and standard deviation in knowledge, attitude, and practice section (Dhaka, Bangladesh. 2021).

There were found to be positive correlations in the KAP domain (Table 3). Knowledge, attitude, and practices correlate significantly (*p* < 0.001). Figure 2 shows the mean and standard deviation of overall knowledge, attitude, and practices. 60% of the study group had inadequate preventative measures (0.60 on a scale of 0–1) and knowledge (around 48%) about DF, as shown in Figure 2.

**TABLE 3:** Association in KAP domain (Dhaka, Bangladesh. 2021).

Association	r-value^a^	*p*-value
Knowledge and Attitude	0.279	<0.001
Knowledge and Practice	0.357	<0.001
Attitude and Practice	0.369	<0.001

^a^ r-value = correlation coefficient.

### Determinants of Knowledge

Table 4 presents the findings of the multiple regression analysis. Significant determinants of DF knowledge were marital status, occupation, and information delivery method. The knowledge of married participants is lower than that of unmarried (B = 0.06, 95% CI: 0.01; 0.10) and other (separated, divorced, etc.) (B = 0.12, 95% CI: 0.07; 0.18) individuals. However, working-class people were more knowledgeable than the jobless (B = −0.07, 95% CI: −0.11; −0.04). Respondents who follow people as their source of information demonstrate less knowledge (B = −0.10, 95% CI: −0.14; −0.07) than those who follow electronic media. Social media followers demonstrate greater knowledge (B = 0.16, 95% CI: 0.10; 0.21) than those who follow electronic media for DF information.

**TABLE 4:** Predictors of knowledge, attitude, and practices regarding dengue fever (Dhaka, Bangladesh. 2021).

Characteristics	Model I knowledge		Model II attitude		Model III practice	
	B^a^ (95% CI^b^)	*p*-value	B^a^ (95% CI^b^)	*p*-value	B^a^ (95% CI^b^)	*p*-value
1. Age (year)						
18–25						
26–35	0.00 (−0.04; 0.04)	0.885	0.00 (−0.02; 0.71)	0.656	0.04 (−0.00; 0.08)	0.077
36–45	−0.00 (−0.05; 0.05)	0.985	−0.00 (−0.04; 0.03)	0.692	−0.00 (−0.05; 0.05)	0.986
More than 45	−0.01 (−0.0 8; 0.05)	0.657	0.02 (−0.02; 0.06)	0.388	0.02 (−0.0 4; 0.08)	0.506
2. Gender						
Female						
Male					−0.06 (−0.10; −0.03)	0.000***
3. Marital status						
Married						
Unmarried	0.06 (0.01; 0.10)	0.023*	0.02 (−0.01; 0.05)	0.259	0.05 (0.00; 0.10)	0.029*
Other (Separated, Divorced)	0.12 (0.07; 0.18)	0.000***	0.04 (0.01; 0.07)	0.020*	0.08 (0.03; 0.13)	0.001**
4. Living with family						
Yes						
No	0.02 (−0.03; 0.06)	0.442				
5. Education						
≥ Primary level						
No education	0.67 (−0.01; 0.05)	0.252	−0.04 (−0.06; −0.02)	0.000***	−0.05 (−0.08; −0.02)	0.002**
6. Occupation						
Worker						
No work	−0.07 (−0.11; −0.04)	0.000***				
7. Media used for dengue fever information						
Electronic media (Television, Radio)						
Social media	0.16 (0.10; 0.21)	0.000***	0.01 (−0.02; 0.04)	0.580	0.02 (−0.03; 0.07)	0.413
People (community, family members)	−0.10 (−0.14; −0.07)	0.000***	−0.02 (−0.04; −0.00)	0.040*	−0.06 (−0.09; −0.03)	0.000***
Others	0.13 (0.03; 0.22)	0.009**	0.02 (−0.04; 0.08)	0.601	−0.03 (−0.12; 0.06)	0.547
	^c^R^2^ = 0.20		^c^R^2^ = 0.05		^c^R^2^ = 0.10	

*p < 0.05; **p < 0.01; ***p < 0.001.

^a^ B, beta coefficient.

^b^ CI, confidence interval.

^c^ R^2^, the coefficient of determination.

### Determinants of Attitude

Marital status, level of education, and forms of media for DF information were significant predictors of attitude (Table 4). Married, uneducated (B = 0.67, 95% CI: −0.01; 0.05), and those who followed people (B = −0.10, 95% CI: −0.14; −0.07) as their DF information source had a poorer attitude than those who were separated (B = 0.04, 95% CI: 0.01; 0.07), had a minimal education level, or followed electronic media, respectively.

### Determinants of Practices

Gender, marital status, education, and media types were identified as significant determinants of practices against DF (Table 4). Male (B = −0.06, 95% CI: −0.10; −0.03), those who were married, those who lacked formal education (B = −0.05, 95% CI: −0.08; −0.02), and those who relied on people (B = −0.10, 95% CI: −0.14; −0.07) only as a source of DF data were found to engage in poor practices than female, unmarried (B = 0.05, 95% CI: 0.00; 0.10), other (Separated, Divorced) (B = 0.08, 95% CI: 0.03; 0.13), who had minimal education, and individuals who relied on electronic media for DF information.

## Discussion

According to our knowledge, this is the first study to apply the KAP model to examine DF responses among slum dwellers of Dhaka, Bangladesh, during the COVID-19 pandemic. Due to socioeconomic deprivation, individuals may be uneducated and unaware of DF [33, 48]. However, the overall attitude exhibited by the studied population was better than knowledge and practices. It should be noted that slum dwellers lacked DF precautions in the previous study [48]. This study was conducted when COVID-19 became a major safety concern. Numerous slum dwellers may be suffering from and fearful of contracting an infectious disease such as DF. Many of them were concerned about the DF outbreak control efforts. Nonetheless, they contemplated keeping their DF preventive efforts throughout the crisis. Due to the fact that this study was conducted during the COVID-19 pandemic, it is essential to note that respondents may have had difficulty identifying the fever symptom, which is also characteristic of COVID-19. The circumstance as a whole may impact DF perception. During the pandemic, one web-based study in Dhaka indicated safety concerns over the DF [6].

### KAP Status Regarding Dengue Fever

According to the present study’s findings, slum dwellers lack fundamental DF knowledge. Many did not know that dengue is an infectious disease and its common symptoms. Dengue is an infectious disease caused by any of the four related dengue viruses, and it is spread to people through the bite of an infected *Aedes* species mosquito [49–51]. Symptoms of dengue typically include a high fever, headache, joint and muscle pains, vomiting, and a skin rash; in severe cases, it can lead to serious bleeding, shock, and death [52]. In the case of our previous web-based studies conducted among the general population and university students, we found that many respondents did not know the infectious behavior of the dengue virus [6, 20]. However, slum dwellers have a lower comprehension level than those with greater access to authentic information. Similarly, our study revealed that many slum dwellers were unaware of the asymptomatic characteristics of DF and that DF can be severe in recurrent infections. Most dengue infections are asymptomatic cases, which cause difficulty in disease control and are essential in dengue surveillance [49]. A second infection with other dengue virus serotypes can be more severe and fatal than the initial infection. In this scenario, dengue hemorrhagic fever and dengue shock syndrome frequently exhibit more severe symptoms, including fever, thrombocytopenia, hemorrhagic signs, and hypovolemia [52]. The existence of cross-reactive antibodies against distinct dengue virus serotypes has been shown to predispose individuals to more severe disease [53]. It leads to the progression of dengue hemorrhagic fever and dengue shock syndrome [54]. These non-neutralizing antibodies can be acquired from a prior infection, passive maternal immunity, or immunization [55]. Our results showed that few individuals correctly identified the risk of dengue virus transmission from pregnant mothers to their fetuses. Bangladesh and Malaysia’s general populations also showed a lack of knowledge regarding the risk of dengue virus transmission during pregnancy [6, 21]. Prior studies have documented this risk [21, 56, 57]. Antibodies against a certain dengue virus serotype might pass the placenta and enter the bloodstream of fetuses, causing a harmful immune response against other serotypes after birth [55]. Children with passive immunity from their mothers are more likely to develop dengue hemorrhagic fever during their initial dengue infection [58, 59]. In addition, many participants failed to correctly identify the breeding locations for *Aedes* mosquitoes, resulting in an increased risk of severe DF, as reported by a previous study [6, 60]. Identifying and eradicating dengue mosquito breeding grounds is essential for reducing the disease’s spread.

Numerous members of the study population agreed on effective efforts to eliminate mosquitoes. However, many did not monitor mosquito eggs and larvae at home and in the environment. Inadequate mosquito breeding prevention techniques have shown a knowledge gap. Additionally, it conforms to research conducted in Bangladesh and Vietnam [6, 38]. *Aedes* mosquitoes breed in water-filled containers such as tires, barrels, plastic drums, jerry cans, and abandoned containers [61, 62]. A study conducted in Malaysia found that many people believed that eliminating dengue mosquito larvae is a complete waste of time [63]. However, these breeding grounds should be identified and eliminated, particularly in tropical and subtropical locations where *Aedes* mosquitoes are prevalent [61]. Moreover, these mosquito breeding sites’ monitoring activities should be maintained even in the off-season when DF is not frequent. Even during the off-season, it is typically advised to continue destroying mosquito breeding sites to avoid the growth of mosquito populations and reduce the risk of disease transmission [64]. Half of our study population also agreed with it. The participants in the survey agreed that fogging alone is insufficient to manage the dengue outbreak; instead, it requires a holistic approach in which families, communities, and the government unite to reduce mosquito breeding sites. This result is consistent with prior research [6, 65]. However, this result contradicts previous research [21, 66], where most communities agreed on chemical fogging and eliminating mosquito breeding areas instead. Chemical fogging is one method for controlling the DF outbreak, with advantages and disadvantages [67]. While chemical fogging efficiently reduces mosquitoes and prevents the spread of diseases transmitted by mosquitoes [68], it can also lead to the development of insecticide resistance, adverse effects on non-target organisms, and potential health risks to humans [69, 70]. In order to effectively manage mosquitoes, it is necessary to use chemical fogging sparingly and in conjunction with other control methods. Few respondents sought fog from the authority, and the majority did not monitor mosquito breeding places. It demonstrated the gap between this group and the authority, highlighted in a recent COVID-19 survey of Bangladeshis [9]. In addition, individuals may feel that authorities will fog the area when necessary. Regular monitoring of mosquito breeding locations is essential, along with aggressive DF control actions [21]. Our research also found that many slum dwellers are unwilling to participate in mosquito control efforts. A study in the slums of Delhi, India, revealed minimal involvement in the local municipality’s mosquito control and health promotion initiatives [71]. They have suggested that participation might result in a favorable shift in behavior. Numerous individuals did not clean the water storage to eliminate mosquito breeding grounds. Dengue-carrying *Aedes* mosquitoes lay their eggs on the walls of water-filled containers in and near homes and terraces [72]. Female mosquitoes may lay eggs up to five times throughout their lifespan, and the eggs can remain viable for months [73]. Identifying and eliminating standing water in and around dwellings is necessary to avoid *Aedes* mosquito breeding [74, 75]. We observed that Dhaka city slum dwellers utilized bed nets to prevent mosquito bites during our further investigation. The WHO recommends maintaining mosquito nets during the day since dengue mosquitoes typically bite during the day [76]. In addition, the study suggests that those who use bed nets to avoid other mosquito-borne diseases may be more cognizant of the danger posed by mosquito bites and may be more vigilant in preventing mosquito bites during the day [27]. Another study found that majority of slum dwellers in Kolkata, India utilized mosquito coils or repellent as a preventative method [77]. For efficient *Aedes* mosquito management, it is necessary to take a multifaceted strategy. A combination of these approaches can aid in reducing mosquitoes and halting the spread of diseases they transmit.

### KAP Determinants

Mortality from dengue can be minimized with early identification based on patient data [39]. Assume that the health workers and organizations working for DF control have access to expedited information, such as the socio-demographic status of individuals as a factor correlating with DF responses. They can intervene and aid in the DF’s effective control attempt in such a circumstance. We observed that a level of education and different media are significant factors in slum dwellers’ DF behavior. In addition, a study conducted in the slums of Kolkata, India, revealed that education significantly influences one’s knowledge and practice [77]. According to studies, improved socioeconomic conditions may contribute to successful DF behavior, including practices [6, 21, 78]. Those without education who follow solely individuals (as opposed to social and electronic media) have weak KAP. Interestingly, married individuals have a lower KAP than single individuals. This result contradicts the prior research done in Malaysia [21]. Although marital status may influence dengue fever knowledge, attitude, and practices, the evidence is limited, and other demographic and socioeconomic factors are likely to have a more substantial effect. Therefore, more study is required to investigate the association between marital status and dengue fever knowledge, attitude, and practices. Without effective DF control initiatives, DF can proliferate in slum areas. Together with national and local authorities, they should organize a committee to perform routine DF surveillance.

### Recommendations

This survey included Dhaka city slum dwellers primarily. This group has been identified as one of the most susceptible to any threat [33, 79]. Therefore, efforts must be made to prepare this population for a DF outbreak. The authority’s plan should ensure proper transmission of knowledge, a positive attitude, and preventative measures regarding DF. Governments must also establish campaigns, social mobilization, and communication to educate and train these dwellers on DF management. Television, radio, and social media may offer educational initiatives, such as short films, case studies, and early warnings on DF outbreak control strategies. A survey in Delhi, India’s urban slums, revealed that television is the primary source of information for DF [80]. In Bangladesh, social media has become an increasingly significant source of news and information [15]. Nonetheless, literacy and cognitive comprehension must be considered while creating and executing these approaches [27]. Through these platforms, the health and disaster management authorities might also include information linked to dengue outbreaks before and after their occurrence. The government must adequately equip its personnel and essential stakeholders to tackle this fatal disease [81]. One study also recommended extensive engagement between researchers, companies, and communities in order to develop efficient dengue management techniques [82]. A study done in the slums of Islamabad, Pakistan, suggests adopting a positive deviance (PD) strategy to enhance the KAP regarding DF [83]. This strategy has enormous potential as a tool for behavior modification and community participation in dengue prevention and management [83]. A small-scale review of malaria research done in Cambodia provided overwhelmingly favorable comments on the PD approach’s efficacy in engaging people [84]. Authorities in Bangladesh may also consider similar method for slum dwellers regarding DF control. A project called “Project Mosblock” has been launched to tackle the ever-growing mosquito-borne disease by giving slum dwellers in Dhaka with a specific sort of curtain [85]. In a pilot study of the initiative, zebra-patterned curtains were supplied to dwellers of one of Bangladesh’s largest slums, the Korail Slum in the city. Officials with the initiative claim that zebra stripes repel bloodsucking insects such as mosquitoes by rendering them incapable of landing on the animal’s skin. Officials of the project thought that the endeavor would curb the mosquito-borne disease outbreak in the slums of the city.

This study’s findings can be applied to other dengue-risk settings, especially informal settlements. Authorities and communities throughout the world are alarmed by the outbreak. Research determined the effect of the COVID-19 pandemic on India’s vector control program [86]. The increased dengue risk caused by the lockdown has been addressed in the previous study [86]. Consequently, assessing the KAP level for DF during the pandemic is imperative. Our KAP model suggests that the increasing knowledge of individuals may result in behavioral changes, such as improved attitudes and practices about DF. In addition, the associated predictors of KAP have been identified. This knowledge might be incorporated into other DF-affected nations’ dengue outbreak control plans, particularly for vulnerable people such as slum dwellers.

### Limitations

The limitations of the current study should be considered when interpreting the results. First, non-probability sampling approaches were utilized during the COVID-19 pandemic. Consequently, this survey may have some inherent biases. Respondents could consider socially acceptable responses, even with the anonymous survey format. So, it might be for the face-to-face interview. Many of the population under study are uneducated and may lack access to certain DF response facilities. Regarding these, respondents exhibited inadequate knowledge, attitudes, and practices. Second, it is considered just the capital city of Bangladesh most severely hit by DF; hence the research population may not represent Bangladesh’s vast population. This exploratory study might offer the Bangladeshi government crucial information despite its limitations. In addition, this study’s insights can aid other impacted areas in integrating DF control efforts, particularly during pandemics.

### Conclusion

During this study, all countries were concerned with pandemic control. Consequently, it may be difficult to concentrate on DF control activities. Nonetheless, the risk of DF can be reduced even during a pandemic by adhering to community standards and adopting traditional wisdom, national legislation, and international standards with the proper authority. Our findings indicate that slum dwellers lacked the knowledge and practices necessary to reduce DF risk. In addition, we may assume that effective dissemination of knowledge, community stimulation toward a positive attitude, and frequent monitoring of preventative activities will be required to control DF outbreak. Increasing the dwellers’ quality of life may also help manage the DF. Therefore, a multidisciplinary strategy is required to influence the behavior of dwellers. It is possible by unifying stakeholders behind a common objective. In order to reduce vector breeding grounds without harming the environment, coupling vector control strategies will rely heavily on individual and community efforts.
